# Modelling variability in cardiac electrophysiology: a moment-matching approach

**DOI:** 10.1098/rsif.2017.0238

**Published:** 2017-08-23

**Authors:** Eliott Tixier, Damiano Lombardi, Blanca Rodriguez, Jean-Frédéric Gerbeau

**Affiliations:** 1 Sorbonne Universités, UPMC Université Paris 6, UMR 7598 LJLL, 75005 Paris, France; 2 Inria Paris, 75012 Paris, France; 3 Department of Computer Science, BHF Centre of Research Excellence, University of Oxford, Oxford, UK

**Keywords:** electrophysiology, moment matching, statistical inverse problems, probability density function, cardiomyocyte ionic channels

## Abstract

The variability observed in action potential (AP) cardiomyocyte measurements is the consequence of many different sources of randomness. Often ignored, this variability may be studied to gain insight into the cell ionic properties. In this paper, we focus on the study of ionic channel conductances and describe a methodology to estimate their probability density function (PDF) from AP recordings. The method relies on the matching of observable statistical moments and on the maximum entropy principle. We present four case studies using synthetic and sets of experimental AP measurements from human and canine cardiomyocytes. In each case, the proposed methodology is applied to infer the PDF of key conductances from the exhibited variability. The estimated PDFs are discussed and, when possible, compared to the true distributions. We conclude that it is possible to extract relevant information from the variability in AP measurements and discuss the limitations and possible implications of the proposed approach.

## 1. Introduction

The variability observed in action potential (AP) measurements is, like in most biological systems, the consequence of many different sources of randomness. In this paper, we focus on parameter randomness which, in the context of AP modelling, corresponds to the natural variability of the cardiomyocyte electrical properties such as its capacitance, ionic channel conductances and gate time constants. Owing to the large number of free parameters in AP models, these parameters are, in practice, unidentifiable [1,2]. In fact, different combinations of these parameters can lead to the same AP. Therefore, we choose to restrict our analysis to ionic channel maximal current densities which, for convenience, are referred to as conductances in the following. Among these conductances, a subset is selected to account for the observed variability depending on the available dataset. AP measurements may result from heterogeneity within a population of cells (inter-subject variability) [3] or from dynamic variations within a single cell (intra-subject variability) [4,5]. In this paper, we propose a novel way to study the variability of parameters of AP model in both contexts. From a modelling point of view, it is convenient to ignore the variability of electrophysiology measurements (and therefore of the underlying parameters) since a set of fixed parameters is sought. However, investigating the variability of parameters of the AP model has several motivations. It can be used to predict the response of cardiomyocytes to certain drugs [6]. It can also provide insight into cell modifications at the origin of common heart diseases such as atrial fibrillation (AF) [3,7] or ventricular arrythmia [8].

There are two main strategies to estimate the parameters' variability given a set of AP measurements. First, one could fit the AP model to each measurement individually and therefore obtain a set of parameters from which useful statistics may be computed. The problem of fitting an individual AP has been addressed many times using a large variety of methods [2,9–13]. However, the computational cost of such a strategy scales with the number of available experimental samples and may therefore be prohibitive. As a consequence, only a low number of cells can be analysed this way. The second strategy belongs to the so-called population of models approach. The experimental set is considered as a whole and the parameters statistics are estimated by solving a statistical inverse problem. Several techniques were developed to solve such problems [14,15] and their application to electrophysiology has recently generated much interest [3,6,16–18]. The present approach belongs to the second strategy. The AP model parameters are described as random variables associated with an unknown probability density function (PDF). The proposed method aims at estimating the parameters PDF, thus generalizing the commonly used mean ± standard deviation intervals. The PDF is sought so that it ‘explains’ the observed variability featured by a given set of AP measurements. More precisely, the estimated PDF is the solution of a constrained optimization problem which is an adaptation of the maximum entropy principle [19]. The method, later referred to as observable moment matching (OMM), is detailed in [20]. Contrary to other approaches such as Monte–Carlo Markov Chains (MCMC) [21] or Approximate Bayesian computation [22], the present method does not guarantee to converge to the true parameters distribution. Instead, it proposes a way to obtain an approximation of the underlying PDF at the fraction of the cost of other finer methods. In this paper, the OMM method is applied to the estimation of the PDF of key conductances from AP measurements. These measurements may be the AP time series (sometimes referred to as waveforms or traces) or be in the form of biomarkers, i.e. features extracted from the time series. Four different case studies are presented to illustrate the use of the OMM method in different scenarios. Test Cases 1 and 2 feature synthetic datasets with AP biomarkers and time series. Test Case 3 features an experimental dataset with intra-subject variability and Test Case 4 features an experimental dataset with inter-subject variability.

## 2. Material and methods

### 2.1. Electrophysiology measurements

#### 2.1.1. Synthetic datasets

For validation purposes, the proposed method is first applied to synthetic measurements, i.e. APs generated by a computational model and corrupted by some noise to solve our statistical inverse problem. An example of such synthetic measurements is shown in figure 2. Here, the noise is an independent zero-mean normally distributed random variable. The signal-to-noise ratio (SNR) is written in dB and defined as follows:
2.1
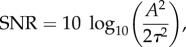
where *τ* is the noise standard deviation and *A* is the AP amplitude.

In Test Cases 1 and 2, the synthetic datasets are generated by evaluating the AP computational model for different values of the parameters, i.e. conductances, of interest. The parameters are sampled from a known distribution so that the estimated PDF may be compared to the true one.

#### 2.1.2. Experimental datasets

In what follows, we are using published AP recordings that are readily available online. In Test Case 3, the experimental dataset consists of several APs recorded on a single canine ventricular cell [4].^1^ This allows us to investigate beat-to-beat variability which is a type of intra-subject variability. About 570 cycles are available, 200 in control conditions and the remaining after the addition of a drug and the modification of the bath ionic centrations. In Test Case 4, the experimental dataset consists of measurements human atrial cardiomyocytes coming from different subjects [3].^2^ Interestingly, the dataset is divided into two groups: one counting 254 sinus rythm (SR) patients and another one counting 215 chronic AF patients.

### 2.2. Electrophysiology cell models

#### 2.2.1. Cell models

Throughout the four test cases presented in this paper, three different AP computational models are used. Using different models serves two purposes. First, it illustrates the fact that the OMM method can successfully be applied to different scenarios. Different cardiac cellular models were used to illustrate that our methods are not model specific. Second, it is more natural to use models that were designed from experimental set-ups that are close to those of the available datasets. In Test Cases 2 and 4, the human atrial model by Courtemanche *et al.* [23] was used. It is one of the first human heart cell models. Mostly based on the Luo & Rudy [24] membrane currents formulations, it was developed using experimental recordings from human atrial cells. In Test Case 1 (respectively, 3), the canine ventricular model by Decker *et al.* [25] (respectively, Davies *et al.* [26]) was used. Both models are improvements of the Hund & Rudy model [27] with updated current formulations to fit canine epicardium (for the Decker model) and mid-myocardial (for the Davies model) cells. All three models belong to the so-called second generation [28] for they provide detailed descriptions of the main ionic channels, pumps and exchangers as well as the internal calcium dynamics. For the sake of convenience, these models will be referred to by their first author's name. We will focus on the PDF estimation of six key conductances corresponding to the following currents: the fast sodium current *I*_Na_, the inward rectifier potassium current *I*_K1_, the transient outward potassium current *I*_to_ (*I*_to1_ in the canine models), the rapid (respectively, slow) delayed rectifier potassium current *I*_Kr_ (respectively, *I*_Ks_) and the L-type calcium current *I*_CaL_. For the sake of clarity, *g*_Na_, *g*_K1_, *g*_to_, *g*_Kr_, *g*_Ks_ and *g*_CaL_ will refer to a multiplicative coefficient for the corresponding values found in the literature. For instance, *g*_Na_ = 1 means that *g*_Na_ is set to the same value as that of the original paper. When necessary, a table will summarize the conductances that have been modified from their reference values.

#### 2.2.2. Numerical methods

The previously mentioned models consist of a set of coupled ordinary differential equations (ODEs) whose formulae are detailed, e.g. on the CellML project website [29]. The Courtemanche and Davies models were implemented in an in-house C++ code and the simulation outputs were compared with those of the Matlab implementations found on the CellML website. The time integration of the ODEs is carried out using the CVODE library [30], which implements the Backward Differentiation Formulae. This state-of-the-art time integrator is well suited to stiff problems as those encountered in electrophysiology. It is adaptive, in time step and order, which can significantly save computational time. For all the test cases, the absolute and relative tolerances of the CVODE solver were set to 10^−6^. For the Decker model, the time integration was carried out using variable but non-adaptive time steps. The stimulation protocol consists in stimulating at a frequency of 1 Hz (or 2 Hz for Test Case 2) over a few cycles so that the recorded AP lies in a permanent regime. In practice, the number of these transition cycles was set to 5 (10 for APs stimulated at 2 Hz) and the relative difference norm between two consecutives AP is less than 0.1%. Unless stated otherwise, the stimulation duration is set to 2 ms and its amplitude to 20 μA.

#### 2.2.3. Action potential time series

In Test Case 1, the AP time series are used as the observable. This means that the inverse procedure possibly uses the AP value at every available time step. This has not only the advantage of capturing all of the available information but also the disadvantage of increasing the computational cost of the inverse procedure because the number of time steps may be large. To tackle this issue, a time-step selection algorithm was developed and is described in [20]. It uses the pre-computed simulation database to approximate the sensitivities with respect to each parameter and for each time step. Using these sensitivities, the time steps are clustered using an agglomerative clustering algorithm and a representative is chosen for each cluster. Only the representatives are retained for the inverse procedure. In practice their number is much lower than the total number of time steps, thus alleviating the computational cost of the inverse procedure. Indeed, as described in [20], the OMM procedure cost is dominated by the inversion of a dense matrix of size (*N*_m_ × *N*_t_)^2^. Furthermore, reducing the number of time steps is motivated by numerical considerations because the conditionning of this matrix deteriorates as the number of time steps increases. This time-step selection comes at no cost because it uses the already computed simulation database.

As the ODEs are solved using an adaptive time-stepping, each AP simulation is discretized on a different time grid and later interpolated on a common grid. This interpolation procedure introduces a numerical error which may be considered as a numerical noise, alongside the noise in the measurements (whether synthetic or experimental).

#### 2.2.4. Action potential biomarkers

**Figure 1.**
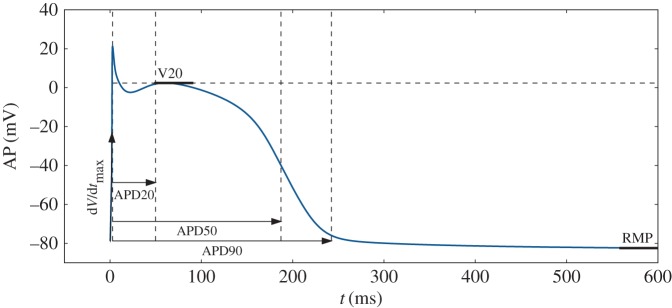
Biomarkers computed from an AP. (Online version in colour.)

In Test Cases 2, 3 and 4, the inverse procedure is applied to so-called biomarkers, which are quantities computed from the AP time series. They describe the main features of the AP such as its shape or its duration. We will focus on the following biomarkers (figure 1): the AP duration APD90 (respectively, APD50, APD30 etc.) at 90% repolarization (respectively 50%, 30% etc.), the resting membrane potential (RMP), the maximum upstroke velocity d*V*/d*t*_max_, the AP value at 20% repolarization V20 (which roughly corresponds to the AP plateau value), the AP value 30 ms after depolarization *V*_notch_ and the area under the curve (AUC), i.e. the AP time integral over one cycle.

Even though these quantities seem to capture well the main features of a given AP, they generally do not convey enough information about the underlying parameters for the inverse procedure. Therefore, the pairwise products (APD90 × APD50, APD90 × APD20 and so on) of the above biomarkers are added to the constraints of moments. Note that the AP triangulation is a commonly used biomarker and may be interpreted as the pairwise product between APD90 and 1/APD30. For the synthetic measurements, the noise is added to the AP time series before computing the biomarkers.

#### 2.2.5. Parameter calibration

We restrain our parameter estimation study to three to six conductances. This assumption is critical and is discussed in the Discussion section. This means that the parameters of interest are allowed to vary, while all the other parameters of the model remain fixed. While it seems reasonable to choose the values found in the literature for these parameters, it often proves to be a bad choice when dealing with real data. Therefore, one needs to calibrate these parameters before performing the inverse procedure using the most representative experimental sample of the available data set. In Test Case 3, the most representative sample is the one whose biomarkers are the closest to the median values (there is one representative for each group). In Test Case 4, the most representative sample is the AP whose APD90 is the closest to the median value. Once these representative samples are identified, a parameter calibration procedure is performed for all ionic conductances. Electronic supplementary material, table S9, shows the values obtained from the literature for these parameters as well as their estimated counterparts for both Courtemanche and Davies models. The table also shows concentrations of some external ion. These were directly set using the bath descriptions available in the publications associated with the experimental datasets. The parameter calibration is actually a constrained minimization problem where the cost function *J* to be minimized reads2.2
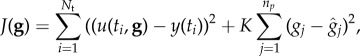
where *y* is the experimental quantity of interest, *u* the corresponding simulation output and *N*_t_ the number of values to be fitted (number of biomarkers or number of time steps depending on the test case). The second part of the cost function is a regularization term, where *n*_*p*_ is the number of conductances to fit, *g*_*j*_ the estimated value of the *j*th conductance, 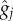 its nominal value and *K* is a user-defined regularization parameter. This term ensures that the conductances remain within a reasonable range around the nominal values. In practice, this parameter is chosen to be small compared to the first term in equation (2.2) so that the conductances are weakly constrained around relevant values without too much impact on the fitting quality. When possible, this parameter *K* may even be set to zero. In the electronic supplementary material, figure S1, a brief study of the effect of *K* is performed. The models considered in this work are not well suited to classical gradient optimization techniques, as they consist of many and strongly nonlinear ODEs, making the gradient computations challenging and the cost function highly irregular. For the sake of simplicity, we, therefore, used gradient-free optimization techniques such as genetic algorithms [10]. We chose the covariance matrix adaptation evolution strategy (CMA-ES) evolutionary algorithm [31], for it is currently one of the most performant genetic algorithms and was used successfully in a variety of applications. Furthermore, a Python (as well as other languages) implementation of the CMA-ES algorithm is available online^3^ and behaves like a black-box optimization tool. The CMA-ES algorithm was recently used in a similar context in [4], where conductances of several models (including the Davies model) were estimated from both synthetic and experimental measurements. Note that values of all parameters are not allowed to take negative values, but they are not limited by any upper bound. An exception is made for the fast sodium conductance *g*_Na_ (which is limited to five times its nominal value) for numerical reasons. Indeed, a high value of *g*_Na_ may lead to a failure of the time integration around the upstroke.

### 2.3. Observable moment matching method

We now give an overview of the OMM method. This method aims at obtaining an approximation of the parameters PDF at a low computational cost. This approximation is, however, not meant to reach the precision of finer methods such as MCMC. The interested reader is referred to [20] where more details are provided.

#### 2.3.1. Construction of the simulation database

The OMM method relies on the pre-computation of a simulation database of many APs (or AP biomarkers) by varying the parameters of interest. We introduce the parameter space *Θ*, which is a subset of 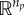 where *n*_*p*_ is the number of parameters (the conductances in our case). A point in *Θ*, or sample of parameters, is denoted by ***θ*** = (*θ*_1_, …, *θ*_*n*_*p*__). The parameter space is discretized using the Sobol sequence [32]. This sampling method is well suited to the present framework: it uniformly spans the parameter space in a low-discrepancy manner while featuring a simple Monte–Carlo quadrature rule; it requires little knowledge of the true parameters distribution; furthermore, as the latin hypercube method used in [6], it only requires a lower and upper bound for each parameter and the total number of samples. Points in the discretized space will be called collocation points and the total number of these points will be denoted by *N*_c_. The discretization of the parameter space is, therefore, given by the set {***θ***_1_, …, ***θ***_*N*_c__}. For each collocation point, one AP is simulated using the numerical protocol described above and stored. Note that once this simulation database is built, no additional AP simulation is required during the inverse procedure.

#### 2.3.2. Optimization problem

Given a PDF *ρ*, the moment of order *m* of the simulations at a given point *t* (time step or biomarker index) is defined by
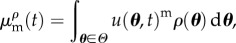
where *u*(***θ***, *t*) is the simulation output, already computed and stored in the database. The empirical moments of order *m* of the measurements at a given point *t* are defined by
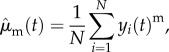
where *y*_*i*_(*t*) is the observable value at point *t* of the *i*th experimental sample and *N* is the total number of experimental samples. The goal of the OMM method is to find the PDF *ρ* such that the moments, up to a certain order *N*_m_, of the simulations and of the experiments match at every point *t*. This moment-matching condition will later be referred to as the moment-matching constraints. As explained above, in the case where many observable quantities are available, a procedure has been set up to select a subset for which the moment-matching constraints hold. In general, however, the stated moment problem is under-determined, meaning there exists an infinity of different *ρ* that satisfies the moment constraints. We propose to regularize the problem using the maximum entropy principle where the entropy of a given PDF *ρ* is given by
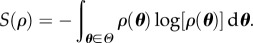
This type of regularization roots in information theory [19], it is considered the most natural choice when limited information about a PDF is available. It is also well suited to our optimization problem for practical mathematical reasons. In Section 2.4.2. of [20], we propose an analysis of the error on the PDF estimation made by adopting the maximum entropy regularization. In Prop. 1. of the same paper, it is shown that under certain conditions on the regularity of the observable and identifiability of the parameters, the error on the PDF is bounded. The conditions on the regularity of the observable may not be easy to check formally because of the nonlinearities of the state equations. Nevertheless, for the practical problems considered in this work, they do not seem critical. The condition on the identifiability may also be difficult to assess in general. In our algorithm, the identifiability issues are circumvented by regularizing the Hessian in the optimization problem and by selecting the points in which the moments are matched (see Discussion section). Finally, the estimated PDF is the solution of the following constrained optimization problem:2.3
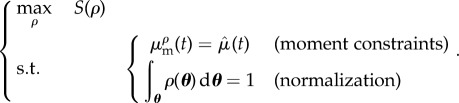
The optimization problem is recast using Lagrange multipliers for the constraints and the corresponding Euler–Lagrange multipliers are solved using a quasi-Newton method. Denoting by |*Θ*| the volume of *Θ*, the integrals over the parameter space of a given quantity *f* are approximated using the Monte–Carlo quadrature rule:2.4
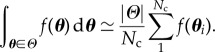
In electronic supplementary material, appendix A, an illustration of the OMM method on a simple test case using the Davies model is provided.

#### 2.3.3. Post-processing

The PDF is a real-valued multivariate function of *n*_*p*_ variables. The output of the OMM method is the estimated PDF values at each collocation point in the parameter space. We insist on the fact that the estimated PDF does not take any parametric form (such as a multivariate Gaussian) but is defined point-wise. However, beyond two dimensions, its visualization becomes complex and may not provide much information. Therefore, as it is the case in the remainder of the article, the PDF is post-processed so that the marginal densities of the parameters may be visualized. The marginal density *z*_*p*_(*x*) of parameter *p* at point *x* reads
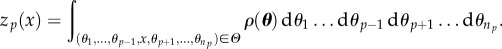
This step actually needs a finer grid in the parameter space than that provided by the Sobol sequence. The estimated PDF is interpolated on the finer grid using kernel smoothing. This step is discussed in detail in [20]. In addition to the marginal densities, the estimated parameter moments *μ*_m_(*θ*_*p*_) may also be computed directly from the PDF:
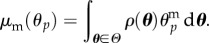
Then, one can compare *μ*_1_(*θ*_*p*_), the mean of parameter *p* and its standard deviation 
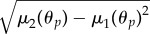
 to their true values when known. In practice, the integral quantities are all approximated using equation (2.4).

#### 2.3.4. Implementation details

An online repository in open access^4^ has been created to make available data and codes used in this paper.

In each test case, the computational time of the inverse procedure is strongly dominated by the simulation database construction. All other steps of the method, including the OMM method itself, have a negligible computational time. The approximative CPU times given for each test case are meant for one processor. This means that the real time may be reduced by simulating the APs in parallel, which is done in practice. Simulations were performed on a Linux machine counting 12 Intel(R) Xeon(R) CPU E5-2640 @ 2.50 GHz processors.

External libraries are used in our code: Eigen 3 and GSL-BLAS for the matrix/vector manipulations and algebra and the Python library Scikit-learn [33] for the time-step selection algorithm.

#### 2.3.5. Comparison with existing methods

As discussed earlier, it is possible to infer the PDF of conductances of interest by performing an individual inverse problem (or fitting) for each sample of the experimental measurements. However, if there are *N* experimental samples, the cost of such an approach would be *N* times the cost of a single fitting. On the contrary, the proposed approach performs the PDF estimation by taking into account only the statistical moments of the set of measurements. Its main advantage is that it does not scale with the number of measurements samples. In that regard, it is, in most scenarios, computationally cheaper than individually estimating the parameters from each sample. Furthermore, all model evaluations are performed offline and once and for all so that the main cost of the inverse procedure can be decided in advance.

Another popular method performing estimations of PDFs is the Bayesian inference. It guarantees to converge to the true PDF, which the present approach does not claim to do, at the expense of many forward model evaluations. The present approach may, therefore, be seen as a less precise but computationally cheaper alternative to Bayesian inference.

In [20], we provide a comparison of our method to two other approaches.

## 3. Results

The observable moment-matching method is now applied to four test cases, using both experimental and synthetic AP measurements.

### 3.1. Test Case 1: Decker model with synthetic data

In this test case, the OMM method is applied to a synthetic dataset using the Decker model with different scenarios: one in control conditions and one with a blocked channel (which models for example the effect of a drug). We show that combining data from both scenarios increases the precision of the PDF estimation of the conductances of interest.

#### 3.1.1. Control conditions

**Figure 2.**
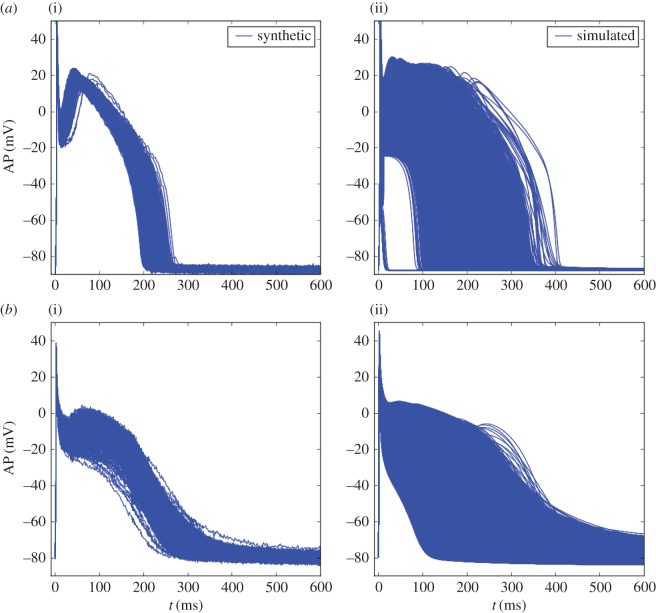
(*a*) AP datasets generated using the Decker model: synthetic data used for the observations (i) and simulation database (ii). (*b*) AP datasets generated using the Courtemanche model: synthetic data used for the observations (i) and simulation database (ii). (Online version in colour.)

For the synthetic dataset, *N* = 10^4^ APs were generated using the Decker model with six uncertain parameters: *g*_Na_, *g*_K1_, *g*_to_, *g*_Kr_, *g*_Ks_ and *g*_CaL_. The *N* samples were drawn from an uncorrelated multivariate normal distribution of mean 1.1 and standard deviation 0.15. The SNR is equal to 41 dB. The simulation database was built by sampling the same six parameters over the domain ***Θ*** = [0.5, 2.0]^6^. *N*_c_ = 2^15^ samples were drawn and the corresponding APs are shown in figure 2. The construction of the simulation database required a CPU time of approximately 1000 min for one processor. For both the synthetic dataset and simulation database, all remaining parameters are fixed and set to their reference values. In this test case, the observable quantities used in the OMM method are the whole AP time series. The observable quantities, are therefore, the AP values at each of the 449 time steps sampled from the time integration grid. The number of moments to be matched is set to *N*_m_ = 3. As mentioned earlier, a procedure has been set up to select only a subset of the available time steps to perform the inverse problem.

**Figure 3.**
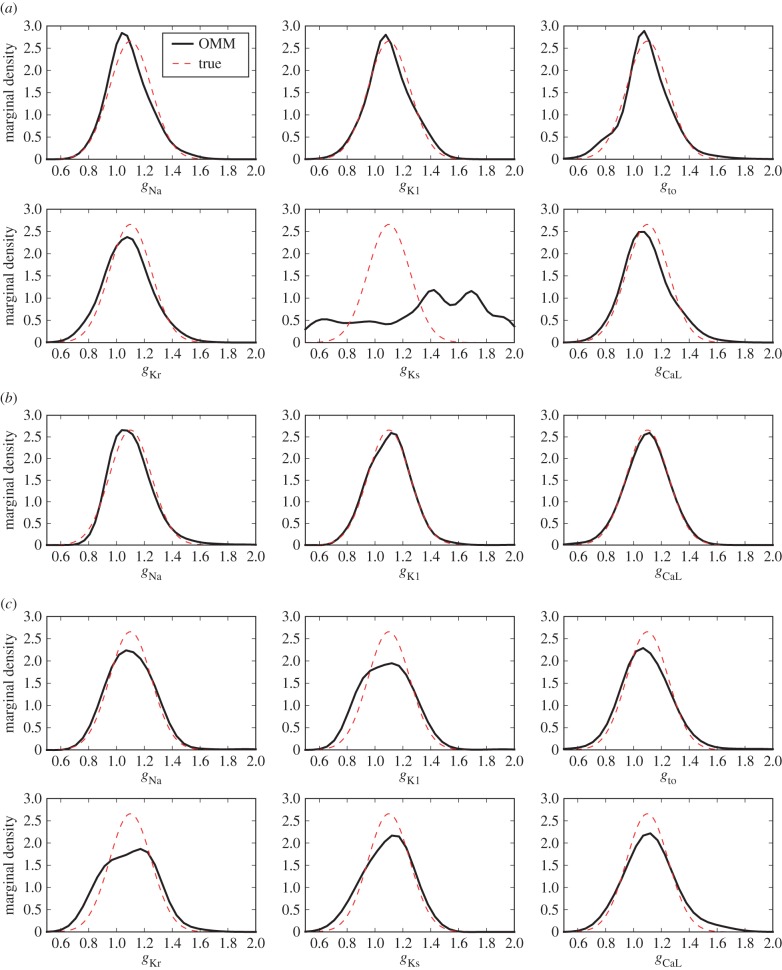
Test Case 1 using the Decker model and synthetic data. Conductances estimated marginal densities (*a*) in control conditions (no drug block), (*b*) in drug block conditions (90% block of *I*_to_, *I*_Kr_ and *I*_CaL_) and (*c*) using combined data from control and drug block conditions. (Online version in colour.)

The OMM method is applied and the resulting estimated marginal densities are shown in figure 3. This allows us to make a clear comparison between the true densities of parameters and their estimated ones. Electronic supplementary material, table S1, shows a more thorough comparison between the statistics of estimated parameters and their true ones. Except for *g*_Ks_, their mean values are accurately estimated (the error is always below 1%) and the errors on the standard deviations range from 3 to 21%. Five out of six conductances are correctly estimated, while the estimation of *g*_Ks_ is poor. This is actually a conductance which is known to be difficult to estimate when others vary, mainly due to the fact that its effect is hidden by other conductances (mainly *g*_Kr_). Therefore, a strategy was devised to reduce the uncertainty on the parameter *g*_Ks_.

#### 3.1.2. Block conditions

To unveil the effects of *g*_Ks_ onto the AP waveform, a drug block scenario is devised to ‘mask’ the effects of the other conductances that compete with *g*_Ks_. Here, we simulate the effect of a hypothetical drug by blocking 90% of the *I*_to_, *I*_Kr_, *I*_CaL_ channels, i.e. by setting the corresponding conductances to 10% of their reference values. The same protocol, as in the control conditions, is followed to generate the synthetic dataset and the simulation database, this time varying only the three remaining conductances (*g*_Na_, *g*_K1_ and *g*_Ks_) with *N* = 10^4^ samples for the synthetic dataset and *N*_c_ = 2^12^ collocation points for the simulation database. The construction of the simulation database required a CPU time of approximately 125 min for one processor.

The OMM method is applied and the results are shown in electronic supplementary material, table S2 and figure 3. The density of *g*_Ks_ is now recovered with a good precision, as the conductances previously responsible for its non-identifiability remain fixed.

#### 3.1.3. Combining control and drug block conditions

The drug block and control conditions are now combined to simultaneously estimate the PDF of the six conductances of interest. This is done by slightly modifying the inverse procedure. In addition to enforcing the moment constraints of the AP values in the control conditions, the moments of the parameters themselves (*g*_Na_, *g*_K1_ and *g*_Ks_) are also constrained to match those estimated in the drug block conditions. In practice, this is easily done by adding these new constraints to the initial set of constraints (see equation (2.3)). This is, therefore, analogous to solving the inverse problem in the control conditions with the additional knowledge of the statistics of parameters obtained in the drug block conditions.

The final results are shown in electronic supplementary material, table S3, and in figure 3. This procedure achieves a much better estimation of the density of *g*_Ks_. The errors on the mean and standard deviation of *g*_Ks_ are significantly reduced while the accuracy of the estimation of the other conductances is similar to that of the control conditions.

### 3.2. Test Case 2: synthetic data at different pacing frequencies

In this test case, the OMM method is applied to a synthetic dataset using the Courtemanche model. Different scenarios are investigated by varying the frequency of the stimulations that trigger the APs.

#### 3.2.1. Control conditions with 1 Hz pacing

**Figure 4.**
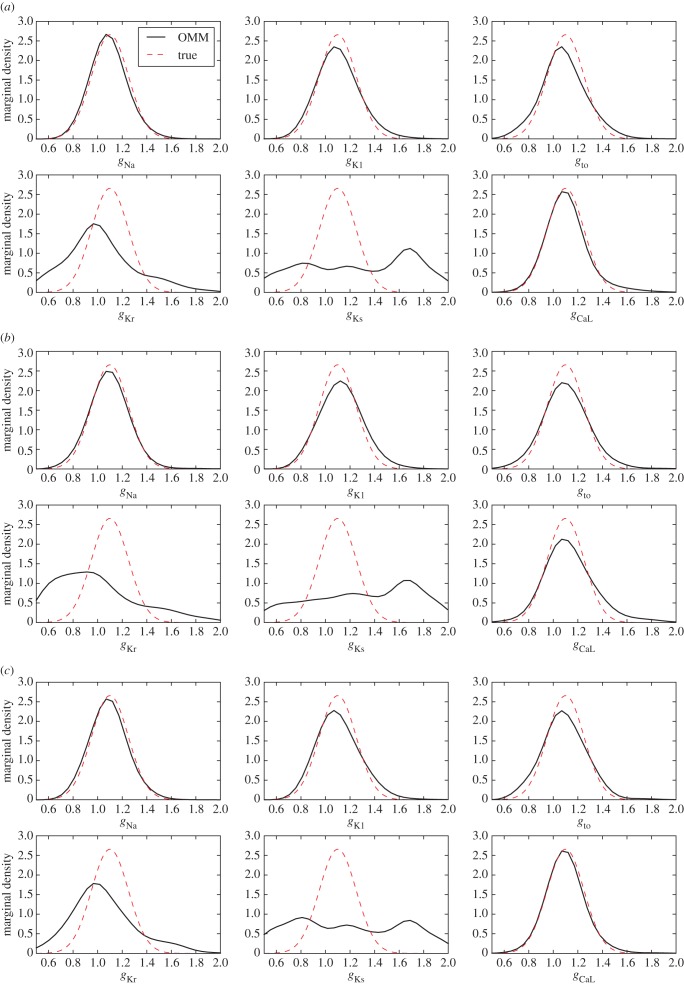
Test Case 2 using the Courtemanche model and synthetic data. Conductances estimated marginal densities in (*a*) control conditions (1 Hz stimulation frequency), (*b*) fast pacing conditions (2 Hz stimulation frequency) and (*c*) with combined data from 1 Hz and 2 Hz pacing. (Online version in colour.)

For the synthetic dataset, *N* = 10^4^ APs were generated using the Decker model with six uncertain parameters: *g*_Na_, *g*_K1_, *g*_to_, *g*_Kr_, *g*_Ks_ and *g*_CaL_. The *N* samples were drawn from an uncorrelated multivariate normal distribution of mean 1.1 and standard deviation 0.15. The SNR is equal to 43 dB. The simulation database was built by sampling the same six parameters over the domain ***Θ*** = [0.5, 2.0]^6^. *N*_c_ = 2^15^ samples were drawn and the corresponding APs are shown in figure 2. The construction of the simulation database required a CPU time of approximately 1100 min for one processor. In this test case, the observable quantities are the following nine AP biomarkers: APD90, APD50, APD30, APA, RMP, V20, d*V*/d*t*_max_, *V*_notch_ and AUC and the maximum moment order is set to *N*_m_ = 2. Adding the pairwise products of biomarkers, the number of moment constraints adds up to 54. The OMM method was applied to this test case and the statistics of estimated parameters are presented in electronic supplementary material, table S4. The estimated marginal densities for each of the six parameters are shown in figure 4. While four out of six conductances are estimated with a reasonable precision, *g*_Kr_, and to greater extent *g*_Ks_, are not well estimated.

#### 3.2.2. 2 Hz pacing

The same simulation protocol is followed, this time by stimulating the APs at a 2 Hz frequency. The accelerated simulation pace induces modifications to the AP morphology (such as a reduced APD) which should reveal new information about the parameters compared to a 1 Hz stimulation. The OMM method was applied to this modified test case. While the exhibited variability differs from the 1 Hz case, no significant improvement over the parameters estimation may be noted. Results are shown in figure 4 and electronic supplementary material, table S5.

#### 3.2.3. Combining 1 Hz and 2 Hz data

A way to take advantage of the information available in the previous two scenarios consists in combining the data obtained at 1 Hz and 2 Hz pacing frequency for both the synthetic dataset and the simulation set. The same inverse procedure, as before, is applied with the following extended set of biomarkers:
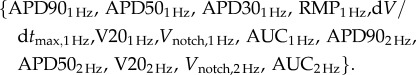
These biomarkers are enriched by their pairwise products which amounts to a total of 119 quantities to be matched. Results are shown in figure 4 and electronic supplementary material, table S6. While *g*_Ks_ is still not correctly estimated, this strategy succeeds in reducing the uncertainty for parameters *g*_Kr_ and *g*_CaL_.

### 3.3. Test Case 3: experimental data from canine ventricular cells

This experimental dataset (used in [4] and available online^5^) features beat-to-beat variability of APs recorded from a single canine ventricular cardiomyocyte. Here, only a subset (traces #100 to #199) of the available dataset is used.

#### 3.3.1. Calibration of the Davies model

**Figure 5.**
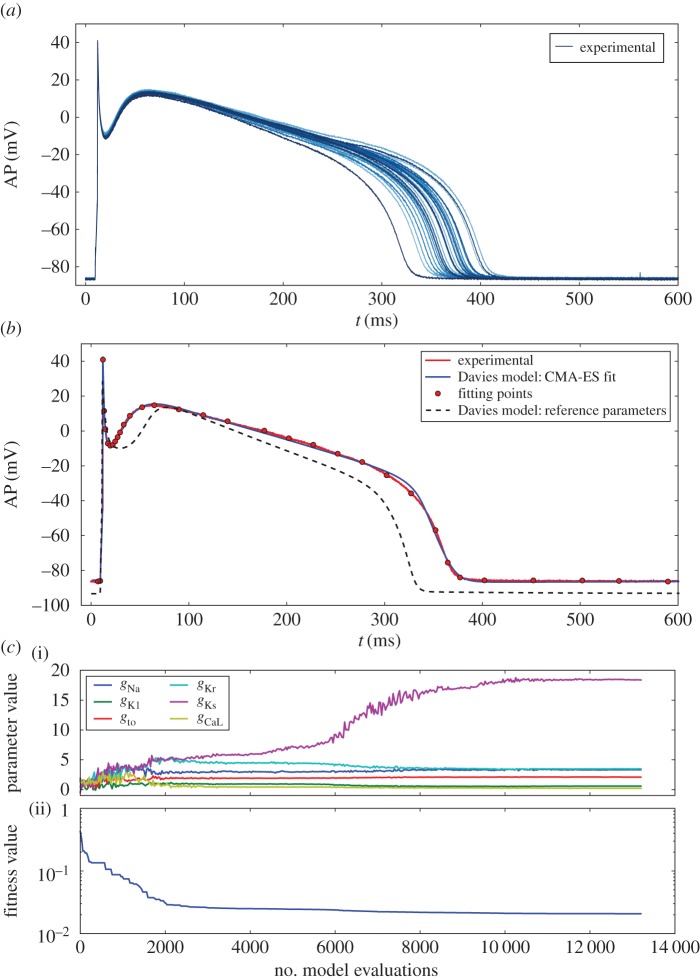
(*a*) Canine ventricular AP experimental set [4]. (*b*) Davies model calibration step: experimental representative AP (solid red), corresponding CMA-ES fit (solid blue) and reference parameters (dashed). (*c*) CMA-ES iterations: (i) values of main conductances are plotted against the number of model evaluations carried out by the CMA-ES algorithm, (ii) corresponding fitness function values.

The Davies model was chosen to study this dataset because it is one of the most recent canine ventricular cell models. In addition, this model was also used in [4] to study the same dataset. The parameter calibration procedure was carried out using the most representative AP of the experimental set and a regularization parameter *K* = 0 (i.e. no regularization). Figure 5 shows the representative AP and its fitted counterpart using the Davies model. In figure 5 is plotted the history of values of six conductances for each iteration of the CMA-ES algorithm. The conductances are normalized with respect to the values found in the reference paper. Note that the values obtained after the calibration are far from the reference values (equal to one by definition), confirming the necessity of such a procedure. This is also true for the other fitted parameters which are not shown in the figure for the sake of clarity but whose values are given in electronic supplementary material, table S7. Note that *g*_Ks_ seems to reach an extremely high value. It is, however, consistent with the values found in [4] and may be explained by a difference in the experimental settings.

#### 3.3.2. Inverse procedure

**Figure 6.**
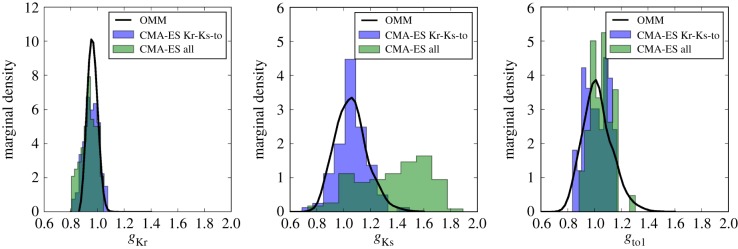
Test Case 3 using the Davies model and 100 AP recordings from a canine ventricular cardiomyocyte. Marginal densities of conductances estimated using the OMM method (solid lines) and individual CMA-ES fits (blue and green bins). Conductances are normalized by the calibrated values.

The OMM procedure is applied with the following biomarkers as observable quantities: APD90, APD50 and *V*_notch_. Here, *V*_notch_ is the notch potential corresponding to the AP value 8 ms after the depolarization peak. *V*_notch_ was preferred over previously introduced V20 because the latter was not suited to the AP shape and its value was almost constant over the experimental set. We made the assumption that the observed variability was due to the variations of *g*_Kr_, *g*_Ks_ (commonly associated with APD variations) and *g*_to1_ (commonly associated with variations of *V*_notch_). These conductances are among the most responsible for beat-to-beat variability [34]. The simulation database was built by sampling these three conductances over the domain ***Θ*** = [0.4, 1.8]^3^ and *N*_c_ = 2^13^ samples were drawn. The construction of the simulation database required a CPU time of approximately 175 min for one processor. The marginal distributions of the three parameters of interest are shown in figure 6 and the estimated statistics are summarized in electronic supplementary material, table S7.

#### 3.3.3. Comparison with individually fitted action potentials

As the exact distributions of the parameters of interest are unknown, a comparison study is carried out using two other PDF estimation techniques. The experimental APs are individually fitted to the Davies model using the CMA-ES algorithm. The same fitting procedure is used as in the calibration step using the AP values at different times (figure 5). In the first case, only the three conductances of interest are allowed to vary, while the others remain fixed. In the second case, all conductances (those concerned by the calibration step) are allowed to vary. In both cases, the fitting procedure yields a collection of *N* = 100 values for the three conductances of interest. The distributions are then approximated using histograms, shown in figure 6. Even though biomarkers were used for the OMM procedure and time series were used for both individual fitting procedures, the distributions of parameters show a striking similarity, especially for the case where only the three conductances of interest are allowed to vary. This suggests that the set of biomarkers retained is enough to account for the observed variability. This also shows the overall satisfactory performances of the observable moment-matching method which achieves comparable results to individual CMA-ES fits at a fraction of the computational cost. Indeed, the 100 individual CMA-ES fits required around 10^5^ model evalutions, while the OMM method required only 8192.

### 3.4. Test Case 4: experimental data from human atrial cells

This experimental dataset (used in [3,35] and available online^6^) features AP biomarkers recorded from two populations of human atrial cells. The OMM procedure is independently applied to both groups and the distributions of the conductances of interest between the two groups are compared.

#### 3.4.1. Human biomarkers dataset

The dataset consists of 469 experimentally recorded sets of seven human AP biomarkers divided in two groups: SR with 254 samples and chronic AF with 215 samples. Both groups exhibit a strong inter-subject variability in addition to the inter-group variability. The available biomarkers are APD90, APD50, APD20, APA, RMP, d*V*/d*t*_max_ and V20.

#### 3.4.2. Courtemanche model calibration

**Figure 7.**
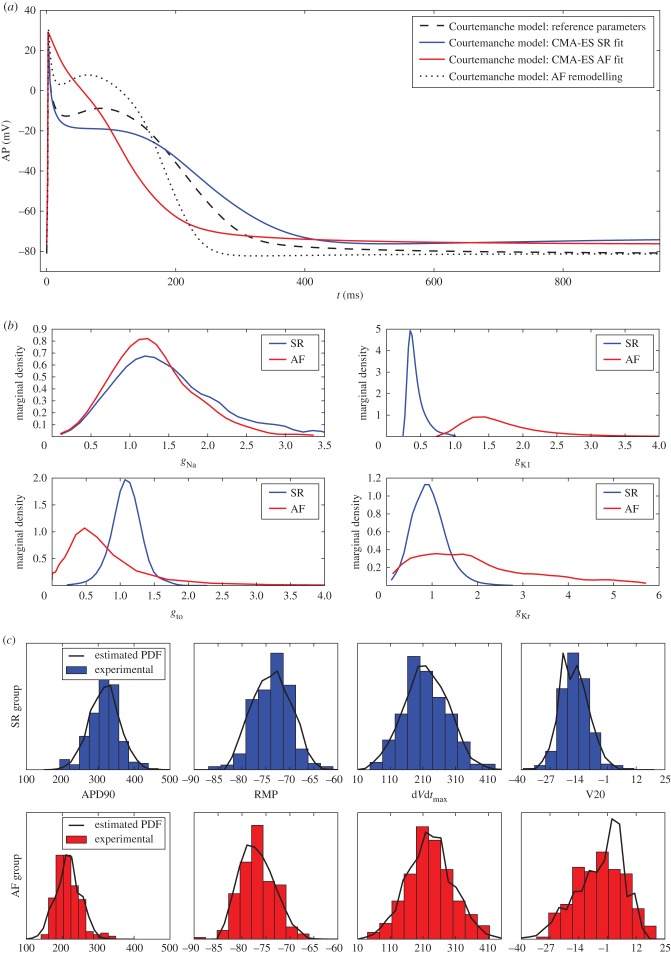
(*a*) CMA-ES parameter calibration of the Courtemanche model prior to the inverse procedure. APs obtained for the most representative samples of the SR (blue) and AF (red) groups, with the reference parameters (dashed) and after AF remodelling (dotted). (*b*) Courtemanche conductances estimated marginal densities for the SR group (blue) and AF group (red). Conductances are normalized by the literature values. (*c*) Normalized histograms of the four experimental biomarkers of interest for both SR (blue) and AF (red) groups. The black solid lines correspond to the PDF of each biomarker estimated by the observable moment matching method.

The Courtemanche model was chosen to study this dataset. Prior to the inverse procedure, a model calibration step is independently carried out for both groups. The regularization parameter is set to *K* = 5 × 10^−3^. The CMA-ES algorithm is applied to fit the Courtemanche model parameters to the most representative sample within each group. The representative sample is the one which minimizes its Euclidean distance to the median biomarkers values of its group. Electronic supplementary material, table S8, shows the most representative samples from each group as well as some global statistics of the biomarkers set. In electronic supplementary material, table S9 are displayed 11 conductances of the Courtemanche model that were estimated during the calibration step. First, for both groups, the values of estimated parameters differ from those found in the literature. Second, there is a significant increase in *g*_K1_ and a significant decrease in *g*_to_, *g*_CaL_ and *g*_Kur_ from the SR to the AF group. These modifications are commonly considered as a good AF remodelling strategy [36–38]. For each set of estimated conductances, an AP is simulated using the Courtemanche model. One obtains a typical or most representative AP for each group. Figure 7 shows such APs along with the AP obtained with the reference parameters found in the literature. The AF AP features a shorter APD and a more triangular shape than the SR one, which is typical of AF [39,40]. This figure also highlights the fact that choosing the literature values as baseline may not be a good choice for the SR group, and to a greater extent for the AF group. In the same figure is added an AP that was obtained by applying the suggested AF remodelling found in [38] to the SR model (65% decrease of *g*_CaL_ and *g*_to_, 49% decrease of *g*_Kur_ and 110% increase of *g*_K1_). Both AF APs are very different and this suggests that AF remodelling should be designed specifically for a given experimental set.

#### 3.4.3. Inverse procedure

The OMM method is applied with four biomarkers of interest as observable quantities: APD90, RMP, d*V*/d*t*_max_ and V20. For each group, a simulation database is built by sampling the following four conductances: *g*_Na_, *g*_K1_ and *g*_to_ and *g*_Kr_. *N*_c_ = 2^14^ samples are drawn using the Sobol sequence and the number of moments to be matched is set to *N*_m_ = 2. The construction of the simulation database required, for each group, a CPU time of approximately 550 min for one processor. The results of the inverse procedure are presented in electronic supplementary material, table S10 and figure 7. As no exact solution is known, one may only qualitatively interpret the result. While *g*_Na_ follows a common distribution in both groups, the other three conductances show striking differences. One way to validate the results is to compare the estimated PDF of the obervable quantities (the four biomarkers of interest) with the experimental one. By construction, they must have the same mean and standard deviation because two moments are matched for each biomarkers. However, this does not guarantee that the distributions are identical because an infinite number of distributions satisfy the moment constraints. In figure 7 are plotted the histograms of the experimental biomarkers along with the estimated biomarkers PDF obtained with the OMM method. In electronic supplementary material, figure S2, we replicated the same plot for the pairwise products of the biomarkers. The distributions are very similar for each biomarkers which suggests that chossing *N*_m_ = 2 is sufficient in this particular case. Note also that, even though the distributions of biomarkers are close to Gaussian ones, this is definitely not true for the distributions of conductances.

## 4. Discussion

In this study, we have presented the OMM method which serves the general purpose of estimating the PDF of uncertain model parameters from a set of measurements. It has been applied to electrophysiology measurements and illustrated with four different test cases.

Test Cases 1 and 2 illustrate the proposed method with synthetic datasets, which has two advantages. First, one may try configurations of a large variety of parameters which may be difficult or impossible to obtain in experimental conditions. Second, knowing the true distributions of the parameters allows for a thorough evaluation of the estimated parameters PDF. In Test Case 1, the proposed method is applied to synthetic measurements generated from the Decker canine model. The OMM method was applied to estimate the PDF of six uncertain conductances. It showed that the OMM method is able to simultaneously estimate the PDF of several conductances.

The authors stress that the proposed method provides an estimation that is an approximation of the real underlying PDF of the parameters. This approximation is less precise than what would be achievable with finer methods such as Bayesian inference but has the advantage of being computationally less demanding in general. In [20], the authors suggest that the present approach could serve as a prior generator for Bayesian inference.

The quality of the estimation obtained depends on the identifiability of the parameters given the available data. However, if a parameter is poorly identifiable (which is the case of *g*_Ks_ in this particular scenario) or even unidentifiable, the method does not fail owing to the approximation of the Hessian associated with the problem in equation (2.3) and the regularization induced by the choice of a subset of time steps where the moments are matched. In that case, such a parameter is characterized by a flat estimated distribution. In the context of experimental data, a strategy may be set up to assess which parameters of the model are actually identifiable, prior to applying the inverse procedure. Such strategies exist (see e.g. [41]) but were not investigated in the present work. Nevertheless, when faced with an estimated flat distribution for one parameter, it is possible to perform the following numerical experiment to assess whether this parameter is unidentifiable or its PDF is, in fact, uniform. Small perturbations (that conserve the norm and positivity of the PDF) may be added to the estimated PDF along the direction of the seemingly unidentifiable parameter. If the moment constraints are still verified, then it probably means that the parameter is, in fact, unidentifiable. To improve the estimation of the hidden *g*_Ks_ parameter, an artifical drug block remodelling was applied to the Decker model. This drug was designed to block the currents that were responsible for the unidentifiability of *g*_Ks_. This remodelling consisted, in practice, in reducing the values of corresponding parameters to 10% of their nominal values. This strategy proved to significantly improve the estimation of *g*_Ks_ and showed that the OMM has potential applications in two contexts. First, it may be used to infer the effect (or toxicity) of a drug using actual experimental data. Second, it may help gain insight into experimental protocols that can be set up to estimate quantities that are otherwise hidden. These findings must however be mitigated by the fact that, in real cells, it is improbable that a given drug only affects a set of targeted ionic currents. It most probably affects the whole cell kinetics and dynamics, including quantities that were supposed to remain unchanged in our artificial scenario. It is also important to note that some stimulation protocols or drug block experiments are not easily achievable in real experiments. In most cases, especially when using human tissue, it is simply not possible to conduct additional experiments, because the tissue is critical to answer more novel research questions. It is an important practicality that makes recordings using animals different from those possible using human tissue. Nevertheless, it may prove useful to inform novel experiments that can be conducted to reduce the uncertainty in the estimation of profiles of conductances based on successful numerical scenarios.

The OMM method is related to the populations of models (POM) approach but differs from it in certain aspects. Whereas the focus of our method is to approximate parameters distributions, POM studies intend to investigate the implications of potential parameters ranges. It would indeed not be possible to confidently estimate conductances from ranges of AP biomarkers and additional constrains would be required, as shown in our study. Other studies such as [6], and those reviewed by Muszkiewicz *et al.* [42] have triggered important discussions and increased interest in an important area of research that requires diversity of techniques and approaches, as shown here. In this context, our study suggests a new method for PDF estimation that may indeed be very useful for new applications.

In Test Case 2, the OMM method is applied to synthetic measurements generated from the Courtemanche human atrial model. The distribution of six conductances were estimated from AP biomarkers obtained in control conditions. Interestingly, the variability observed in the biomarkers set is less informative than that of the AP traces themselves. This is highlighted by the fact that distributions of two conductances are poorly estimated compared to the first test case. Indeed, the biomarkers are features computed from the AP traces themselves and are therefore doomed to carry as much or less information about the underlying parameters. However, studying biomarkers instead of AP traces is justified by the fact that, in practice, certain experimental sets contain only biomarker values. To tackle this, a strategy was set up to extract more information from the AP biomarkers. This was done by changing the stimulation frequency which unveiled new dynamics and therefore new information about the parameters. Interestingly, such a strategy may easily be transposed to an actual experimental protocol. It is, in fact, commonly practised in cardiomyocyte experimental studies [43]. Combining the data obtained using two different frequencies improved the estimation of *g*_Kr_ and *g*_CaL_. *g*_Ks_ was, however, still poorly estimated, mainly because its effect is very similar to that of *g*_Kr_, with a lower amplitude. The investigation of richer stimulation protocols, such as in [2], in order to improve the estimation of poorly identifiable parameters could be the focus of future investigations. It is, in certain cases, possible to successfully estimate *g*_Ks_ by conducting an adequate numerical experiment. In [4] for instance, the authors use the combined recordings of an AP in normal conditions and with *g*_Ks_ set to zero.

In Test Case 3, the OMM method is applied to a set of experimental canine APs recorded from a single canine ventricular cardiomyocyte. This experimental set is an illustration of beat-to-beat variability which is mostly characterized by variations of the APD. It is, therefore, natural to make the hypothesis that these variations are in fact due to fluctuations in the magnitudes of delayed-rectifier potassium currents (*g*_Kr_ and *g*_Ks_) which are the most responsible for APD variations. The APs also exhibit variations around the notch region which motivated the addition of *g*_to_ as the third uncertain parameter. These conductances are known to be the ones that contribute most to beat-to-beat variability [34]. All the other parameters were set to a fixed value using a calibration procedure. Many values of conductances deviate a lot from their reference values which suggests that this step is necessary prior to any variability study. The estimated PDF shows that the large variability observed in the APD is in fact caused by small variations of the underlying parameters. These findings were confirmed by carrying out two other independent parameter estimations which yielded similar distributions for the conductances of interest. For *g*_Ks_, the distribution differs when all the conductances are allowed to vary. This may be explained by the fact that this parameter is less identifiable compared to *g*_Kr_, so that its effect may be compensated or may interfere with other conductances. Some limitations pertaining to the experimental set must be considered. Indeed, the isolation of cardiomyocytes is known to affect the membrane ionic channels [44] and therefore the distributions obtained for the conductances of interest may not reflect the *in vivo* ones. Furthermore, the experimental traces considered are just a snapshot of the cell at a certain state. Therefore, extrinsic factors operating at a long time scale and contributing to variations of the AP features are neglected. For instance, monitoring the APD over the full experimental set reveals that there are long time scale increasing and decreasing trends in the APD (see the electronic supplementary materials in [4]).

In Test Case 4, the OMM method is applied to an experimental set containg AP biomarkers obtained from two different populations: SR and AF. To each group is associated a most representative individual whose values of biomarkers are the closest to the median ones of its group. The calibration step is very informative as it allows for a first comparison between the two groups, or more precisely between the two representatives of each group. The calibration leads to high differences for *g*_K1_ (+220%), *g*_to_ (−100%), *g*_CaL_ (−63%) and *g*_Kur_ (−60%) which are qualitatively similar to those reported in [3]. These differences between the two groups are also in agreement with the AF remodelling mechanisms documented in [36–38,45]. The role of *I*_Kur_ seems to be prominent in the onset of AF [7] along with perturbations of the intracellular Ca^2+^ dynamics [39] which is coupled to the L-type calcium current *I*_CaL_. Beyond these inter-group variations captured in the calibration step, the inter-group variability is revealed by the study of the estimated PDFs. The results highlight the distribution differences of *g*_to_ and *g*_Kr_ between the two groups. In the SR group, these two conductances feature a normal-like distribution that does not deviate much from the mean value, whereas in the AF group those distributions are skewed and much more spread. The distribution of *g*_Na_ is similar between the two groups, which suggests that it does not play an important role in the AF mechanisms. *g*_K1_ also features a much higher mean value and higher variance in the AF group. A posteriori distributions of the biomarkers of interest may be computed from the estimated PDF. When compared with the actual distributions (approximated by histograms of the experimental biomarkers), it shows that the OMM method succeeded in matching the variability in the measurements. In the future, studying other biomarkers or other types of measurements may lead to a better understanding of the AF mechanisms and of the sources of variability within each group.

We now discuss limitations concerning inverse problems in electrophysiology in general and the OMM method in particular. Akin to many inverse problem studies in electrophysiology, we make the assumption that all variability observed in the experimental dataset can be explained by the variation of only a few conductances. Not only are there a large number of different conductances but there are also other parameters such as the parameters governing the dynamics of the channel gates. However, such a simplification is supported by two main considerations. First, the proposed approach is limited by its computational cost. Considering a large number of free parameters means that more samples are required to span the high-dimensional parameter space, which may be intractable in practice. Second, the information available in the AP traces is not enough to constrain all the model parameters. Adding other sources of information such as intracellular calcium concentrations revealed by fluorescence [46] or cell impedence [47] may allow the estimation of more than six parameters. Considering that, choosing the right set of varying conductances is still paramount.

The rationale for choosing the six conductances investigated in this work was based on their known importance in determining the cardiac AP, and key properties including upstroke velocity, plateau duration, resting potential and AP duration. Among them, we included gKs knowing that due to the redundancy of currents during repolarization it would be expected to be poorly identifiable. Our method can, however, be extended to include variability in additional parameters if needed.

Another limitation comes from the experimental sets themselves. Cells coming from different regions of the heart exhibit different variability patterns in their APs. In the context of assessing the effect of a drug or investigating the causes of a heart disease, this approach should be repeated with a wider variety of cell locations. Furthermore, the electrical behaviour of an isolated cell differs from one that is embedded in a tissue. Therefore, using measurements at the tissue scale [48] (for example using MEA measurements [49]) may yield results that are closer to the *in vivo* conditions.

Another point to be discussed is the use of biomarkers versus time traces. This is often imposed by the type of experimental data available. Ranges of biomarkers using standard protocols are easily accessed by experimentalists, and raw AP data are not always available. It is, therefore, important to evaluate the use of both biomarker ranges and AP traces. The set of available biomarkers is often dictated by experimental constraints. It is, however, possible, when there are many available biomarkers, to conduct a preliminary study to determine which biomarkers should be taken into account in order to recover certain parameters of interest. Such a study would consist in applying the proposed method several times with different underlying variations of parameters. Then, for a given set of experimental constraints, it would be possible to assess whether the proposed method would be able to recover the underlying distributions of parameters. Finally, the choice of numerical settings pertaining to the OMM method is discussed. The OMM method relies on the matching of the statistical moments of some observable quantities. The number of moments *N*_m_ to be matched is therefore important. In most applications, choosing *N*_m_ = 2 or 3 is sufficient to capture the distribution of parameters. A common heuristics is to increase *N*_m_ until no siginificant change in the estimated PDF is observed. Note that using high *N*_m_ often leads to numerical instability, all the more so if the noise level in the measurements is high.

In summary, we have presented a new method for estimating the PDF of parameters of AP models from various sets of AP measurements. The AP measurements may come in the form of waveforms (time series) or biomarkers. The method has been illustrated with both synthetic and experimental sets which exhibit both inter-subject and intra-subject variability. The approach we describe has potentially important implications in drug safety pharmacology and more generally in the understanding of variability in ionic properties of cardiomyocytes. It intends to be in line with recent works, suggesting that computational models are a powerful tool to evaluate drug toxicity [50]. More generally, the proposed approach may be a new way to investigate the sources of variability observed in electrophysiology that are experimentally difficult to assess.

## Supplementary Material

Supplementary Material

## Endnotes

1 Data are available here http://www.cs.ox.ac.uk/chaste/download.html.

2 Data are available here http://journals.plos.org/plosone/article?id=10.1371/journal.pone.0105897.

3 https://www.lri.fr/~hansen/cmaes_inmatlab.html#python.

4 https://github.com/eltix/omm_jrsi.

5 http://www.cs.ox.ac.uk/chaste/download.html.

6 http://journals.plos.org/plosone/article?id=10.1371/journal.pone.0105897.

## Data accessibility

An online repository in open access^7^ has been created to make available data and codes used in this paper.

## References

[1] Sarkar AX, Sobie EA 2010 Regression analysis for constraining free parameters in electrophysiological models of cardiac cells. PLoS Comput. Biol. 6, e1000914 (10.1371/journal.pcbi.1000914) 20824123 PMC2932676

[2] Dokos S, Lovell NH 2004 Parameter estimation in cardiac ionic models. Prog. Biophys. Mol. Biol. 85, 407–431. (10.1016/j.pbiomolbio.2004.02.002) 15142755

[3] Sánchez C, Bueno-Orovio A, Wettwer E, Loose S, Simon J, Ravens U, Pueyo E, Rodriguez B 2014 Inter-subject variability in human atrial action potential in sinus rhythm versus chronic atrial fibrillation. PLoS ONE 9, e105897 (10.1371/journal.pone.0105897) 25157495 PMC4144914

[4] Johnstone RH, Chang ETY, Bardenet R, De Boer TP, Gavaghan DJ, Pathmanathan P, Clayton RH, Mirams GR 2015 Uncertainty and variability in models of the cardiac action potential: can we build trustworthy models? J. Mol. Cell Cardiol. 96, 49–62. (10.1016/j.yjmcc.2015.11.018) 26611884 PMC4915860

[5] Pueyo E *et al.* 2016 Experimentally-based computational investigation into beat-to-beat variability in ventricular repolarization and its response to ionic current inhibition. PLoS ONE 11, e0151461 (10.1371/journal.pone.0151461) 27019293 PMC4809506

[6] Britton OJ, Bueno-Orovio A, Van Ammel K, Lu HR, Towart R, Gallacher DJ, Rodriguez B 2013 Experimentally calibrated population of models predicts and explains intersubject variability in cardiac cellular electrophysiology. Proc. Natl Acad. Sci. USA 110, E2098–E2105. (10.1073/pnas.1304382110) 23690584 PMC3677477

[7] Wettwer E, Hála O, Christ T, Heubach JF, Dobrev D, Knaut M, Varró A, Ravens U 2004 Role of IKur in controlling action potential shape and contractility in the human atrium influence of chronic atrial fibrillation. Circulation 110, 2299–2306. (10.1161/01.CIR.0000145155.60288.71) 15477405

[8] Gemmell P, Burrage K, Rodriguez B, Quinn TA 2014 Population of computational rabbit-specific ventricular action potential models for investigating sources of variability in cellular repolarisation. PLoS ONE 9, e90112 (10.1371/journal.pone.0090112) 24587229 PMC3938586

[9] Hui BBCB, Dokos S, Lovell NH 2007 Parameter identifiability of cardiac ionic models using a novel CellML least squares optimization tool. In 2007 29th Annual Int. Conf. of the IEEE Engineering in Medicine and Biology Society, pp. 5307–5310. New York, NY: IEEE. 10.1109/IEMBS.2007.4353539 18003205

[10] Syed Z, Vigmond E, Nattel S, Leon LJ 2005 Atrial cell action potential parameter fitting using genetic algorithms. Med. Biol. Eng. Comput. 43, 561–571. (10.1007/BF02351029) 16411628

[11] Chen F, Chu A, Yang X, Lei Y, Chu J 2012 Identification of the parameters of the Beeler–Reuter ionic equation with a partially perturbed particle swarm optimization. IEEE Trans. Biomed. Eng. 59, 3412–3421. (10.1109/TBME.2012.2216265) 22955867

[12] Kaur J, Nygren A, Vigmond EJ 2014 Fitting membrane resistance in single cardiac myocytes reduces variability in parameters. In Computing in cardiology 2014 (ed. Murray A), pp. 209–212. New York, NY: IEEE.

[13] Lombardo DM, Fenton FH, Narayan SM, Rappel W-J 2016 Comparison of detailed and simplified models of human atrial myocytes to recapitulate patient specific properties. PLoS Comput. Biol. 12, e1005060 (10.1371/journal.pcbi.1005060) 27494252 PMC4975409

[14] Koutsourelakis P-S 2009 A multi-resolution, non-parametric, Bayesian framework for identification of spatially-varying model parameters. J. Comput. Phys. 228, 6184–6211. (10.1016/j.jcp.2009.05.016)

[15] Grenier E, Louvet V, Vigneaux P 2014 Parameter estimation in non-linear mixed effects models with SAEM algorithm: extension from ode to PDE. ESAIM: Math. Model. Numer. Anal. 48, 1303–1329. (10.1051/m2an/2013140)

[16] Romero L, Pueyo E, Fink M, Rodríguez B 2009 Impact of ionic current variability on human ventricular cellular electrophysiology. Am. J. Physiol. Heart Circ. Physiol. 297, H1436–H1445. (10.1152/ajpheart.00263.2009) 19648254

[17] Marder E, Taylor AL 2011 Multiple models to capture the variability in biological neurons and networks. Nat. Neurosci. 14, 133–138. (10.1038/nn.2735) 21270780 PMC3686573

[18] Drovandi CC, Cusimano N, Psaltis S, Lawson BAJ, Pettitt AN, Burrage P, Burrage K 2016 Sampling methods for exploring between-subject variability in cardiac electrophysiology experiments. J. R. Soc. Interface 13, 20160214 (10.1098/rsif.2016.0214) 27512137 PMC5014056

[19] Jaynes ET 1957 Information theory and statistical mechanics. Phys. Rev. 106, 620–630. (10.1103/PhysRev.106.620)

[20] Gerbeau J-F, Lombardi D, Tixier E 2016 A moment-matching method to study the variability of phenomena described by partial differential equations. See https://hal.archives-ouvertes.fr/hal-01391254.

[21] Rosenthal JS 1995 Minorization conditions and convergence rates for Markov chain Monte Carlo. J. Am. Stat. Assoc. 90, 558–566. (10.1080/01621459.1995.10476548)

[22] Barber S, Voss J, Webster M 2015 The rate of convergence for approximate Bayesian computation. Electron. J. Stat. 9, 80–105. (10.1214/15-EJS988)

[23] Courtemanche M, Ramirez RJ, Nattel S 1998 Ionic mechanisms underlying human atrial action potential properties: insights from a mathematical model. Am. J. Physiol. Heart Circ. Physiol. 275, H301–H321. 10.1152/ajpheart.1998.275.1.H301 9688927

[24] Luo C-H, Rudy Y 1994 A dynamic model of the cardiac ventricular action potential. I. Simulations of ionic currents and concentration changes. Circ. Res. 74, 1071–1096. (10.1161/01.RES.74.6.1071) 7514509

[25] Decker KF, Heijman J, Silva JR, Hund TJ, Rudy Y 2009 Properties and ionic mechanisms of action potential adaptation, restitution, and accommodation in canine epicardium. Am. J. Physiol. Heart Circ. Physiol. 296, H1017–H1026. (10.1152/ajpheart.01216.2008) 19168720 PMC2670702

[26] Davies MR, Mistry HB, Hussein L, Pollard CE, Valentin J-P, Swinton J, Abi-Gerges N 2011 An in silico canine cardiac midmyocardial action potential duration model as a tool for early drug safety assessment. Am. J. Physiol. Heart Circ. Physiol. 302, H1466–H1480. (10.1152/ajpheart.00808.2011) 22198175

[27] Hund TJ, Rudy Y 2004 Rate dependence and regulation of action potential and calcium transient in a canine cardiac ventricular cell model. Circulation 110, 3168–3174. (10.1161/01.CIR.0000147231.69595.D3) 15505083 PMC1851913

[28] Kogan BJ 2009 Introduction to computational cardiology: mathematical modeling and computer simulation. Berlin, Germany: Springer Science & Business Media.

[29] Cuellar AA, Lloyd CM, Nielsen P, Bullivant DP, Nickerson D, Hunter P 2003 An overview of CellML 1.1, a biological model description language. Simulation 79, 740–747. (10.1177/0037549703040939)

[30] Cohen SD, Hindmarsh AC 1996 CVODE, a stiff/nonstiff ODE solver in C. Comput. Phys. 10, 138–143. (10.1063/1.4822377)

[31] Hansen N 2006 The CMA evolution strategy: a comparing review. In Towards a new evolutionary computation (eds Lozano JA, Larrañaga P, Inza I, Bengoetxea E), pp. 75–102. Berlin, Germany: Springer.

[32] Sobol IM 1976 Uniformly distributed sequences with an additional uniform property. USSR Comput. Math. Math. Phys. 16, 236–242. (10.1016/0041-5553(76)90154-3)

[33] Pedregosa F *et al.* 2011 Scikit-learn: machine learning in Python. J. Mach. Learn. Res. 12, 2825–2830.

[34] Pueyo E *et al.* 2016 Experimentally-based computational investigation into beat-to-beat variability in ventricular repolarization and its response to ionic current inhibition. PLoS ONE 11, e0151461 (10.1371/journal.pone.0151461) 27019293 PMC4809506

[35] Ravens U *et al.* 2015 Application of the RIMARC algorithm to a large data set of action potentials and clinical parameters for risk prediction of atrial fibrillation. Med. Biol. Eng. Comput. 53, 263–273. (10.1007/s11517-014-1232-0) 25466224

[36] Dobrev D, Ravens U 2003 Remodeling of cardiomyocyte ion channels in human atrial fibrillation. Basic Res. Cardiol. 98, 137–148. (10.1007/s00395-003-0409-8) 12883831

[37] Christ T, Wettwer E, Voigt N, Hala O, Radicke S, Matschke K, Varro A, Dobrev D, Ravens U 2008 Pathology-specific effects of the IKur/Ito/IK, ACh blocker AVE0118 on ion channels in human chronic atrial fibrillation. Br. J. Pharmacol. 154, 1619–1630. (10.1038/bjp.2008.209) 18536759 PMC2518460

[38] Wilhelms M, Hettmann H, Maleckar MM, Koivumäki JT, Dössel O, Seemann G 2013 Benchmarking electrophysiological models of human atrial myocytes. Front. Physiol. 3, 487 (10.3389/fphys.2012.00487) 23316167 PMC3539682

[39] Van Wagoner DR, Pond AL, Lamorgese M, Rossie SS, McCarthy PM, Nerbonne JM 1999 Atrial L-type Ca^2+^ currents and human atrial fibrillation. Circ. Res. 85, 428–436. (10.1161/01.RES.85.5.428) 10473672

[40] Lee Y-S, Hwang M, Song J-S, Li C, Joung B, Sobie EA, Pak H-N 2016 The contribution of ionic currents to rate-dependent action potential duration and pattern of reentry in a mathematical model of human atrial fibrillation. PLoS ONE 11, e0150779 (10.1371/journal.pone.0150779) 26964092 PMC4795605

[41] Pant S, Lombardi D 2015 An information-theoretic approach to assess practical identifiability of parametric dynamical systems. Math. Biosci. 268, 66–79. (10.1016/j.mbs.2015.08.005) 26292167

[42] Muszkiewicz A *et al.* 2016 Variability in cardiac electrophysiology: using experimentally-calibrated populations of models to move beyond the single virtual physiological human paradigm. Prog. Biophys. Mol. Biol. 120, 115–127. (10.1016/j.pbiomolbio.2015.12.002) 26701222 PMC4821179

[43] Zhou X, Bueno-Orovio A, Orini M, Hanson B, Hayward M, Taggart P, Lambiase PD, Burrage K, Rodriguez B 2016 In vivo and in silico investigation into mechanisms of frequency dependence of repolarization alternans in human ventricular cardiomyocytes. Circ. Res. 118, 266–278. (10.1161/CIRCRESAHA.115.307836) 26602864 PMC4719495

[44] Yue L, Feng J, Li G, Nattel S 1996 Transient outward and delayed rectifier currents in canine atrium: properties and role of isolation methods. Am. J. Physiol. Heart Circ. Physiol. 270, H2157–H2168. 10.1152/ajpheart.1996.270.6.H2157 8764269

[45] Koivumäki JT, Seemann G, Maleckar MM, Tavi P 2014 In silico screening of the key cellular remodeling targets in chronic atrial fibrillation. PLoS Comput. Biol. 10, e1003620 (10.1371/journal.pcbi.1003620) 24853123 PMC4031057

[46] Shinnawi R, Huber I, Maizels L, Shaheen N, Gepstein A, Arbel G, Tijsen AJ, Gepstein L 2015 Monitoring human-induced pluripotent stem cell-derived cardiomyocytes with genetically encoded calcium and voltage fluorescent reporters. Stem Cell Rep. 5, 582–596. (10.1016/j.stemcr.2015.08.009) PMC4624957 26372632

[47] Abassi YA *et al.* 2012 Dynamic monitoring of beating periodicity of stem cell-derived cardiomyocytes as a predictive tool for preclinical safety assessment. Br. J. Pharmacol. 165, 1424–1441. (10.1111/j.1476-5381.2011.01623.x) 21838757 PMC3372727

[48] RH Clayton *et al.* 2011 Models of cardiac tissue electrophysiology: progress, challenges and open questions. Prog. Biophys. Mol. Biol. 104, 22–48. (10.1016/j.pbiomolbio.2010.05.008) 20553746

[49] Clements M, Thomas N 2014 High-throughput multi-parameter profiling of electrophysiological drug effects in human embryonic stem cell derived cardiomyocytes using multi-electrode arrays. Toxicol. Sci. 140, 445–461. (10.1093/toxsci/kfu084) 24812011

[50] Davies MR *et al.* 2016 Recent developments in using mechanistic cardiac modelling for drug safety evaluation. Drug Discov. Today 21, 924–938. (10.1016/j.drudis.2016.02.003) 26891981 PMC4909717

