# Supplementary material for: Modelling variability in cardiac electrophysiology: a moment-matching approach
Source: J R Soc Interface. 2017 Aug 23;14(133):20170238. doi: 10.1098/rsif.2017.0238 (PMC5582121; doi:10.1098/rsif.2017.0238)
Supplement: Supplementary Material [file rsif20170238supp1.pdf]

S1 Supplementary Material for:
Modeling Variability in Cardiac Electrophysiology: A Moment Matching Approach

Eliott Tixier[2,1], Damiano Lombardi[1,2], Blanca Rodriguez[3], Jean-Frédéric Gerbeau[1,2,*]

1 Inria Paris, 75012 Paris, France
2 Sorbonne Universités, UPMC Univ Paris 6, UMR 7598 LJLL, 75005 Paris, France
3 Department of Computer Science, BHF Centre of Research Excellence, University of Oxford, Oxford, United Kingdom

* jean-frederic.gerbeau@inria.fr

Additional tables

Table 1. Statistics summary of the estimated parameters from the Decker model in control conditions (no drug block).

Parameter	μ	μ_{ex}	rel. err.	σ	σ_{ex}	rel. err.
g_{Na}	1.104	1.100	**0.4%**	0.155	0.150	**3.1%**
g_{K1}	1.099	1.100	**0.1%**	0.164	0.150	**9.2%**
g_{to}	1.103	1.100	**0.3%**	0.182	0.150	**21.2%**
g_{Kr}	1.091	1.100	**0.9%**	0.172	0.150	**14.4%**
g_{Ks}	1.264	1.100	**14.9%**	0.426	0.150	**184.0%**
g_{CaL}	1.102	1.100	**0.2%**	0.177	0.150	**17.8%**

Table 2. Statistics summary of the estimated parameters from the Decker model in drug block conditions (90% block for I_{to},I_{Kr} and I_{CaL}).

Parameter	μ	μ_{ex}	rel. err.	σ	σ_{ex}	rel. err.
g_{Na}	1.104	1.100	**0.4%**	0.158	0.150	**5.5%**
g_{K1}	1.102	1.100	**0.1%**	0.154	0.150	**2.6%**
g_{Ks}	1.100	1.100	**0.0%**	0.158	0.150	**5.1%**

Table 3. Statistics summary of the estimated parameters from the Decker model in control conditions with moments constraints from drug block estimation.

Parameter	μ	μ_{ex}	rel. err.	σ	σ_{ex}	rel. err.
g_{Na}	1.104	1.100	**0.4%**	0.158	0.150	**5.5%**
g_{K1}	1.102	1.100	**0.1%**	0.154	0.150	**2.6%**
g_{to}	1.104	1.100	**0.4%**	0.182	0.150	**21.3%**
g_{Kr}	1.101	1.100	**0.1%**	0.173	0.150	**15.6%**
g_{Ks}	1.100	1.100	**0.0%**	0.158	0.150	**5.1%**
g_{CaL}	1.102	1.100	**0.2%**	0.176	0.150	**17.1%**

Table 4. Statistics summary of the estimated parameters from the Courtemanche model in control conditions.

Parameter	μ	μ_{ex}	rel. err.	σ	σ_{ex}	rel. err.
g_{Na}	1.098	1.100	**0.2%**	0.149	0.150	**0.7%**
g_{K1}	1.102	1.100	**0.2%**	0.163	0.150	**9.1%**
g_{to}	1.109	1.100	**0.8%**	0.180	0.150	**20.1%**
g_{Kr}	1.087	1.100	**1.0%**	0.302	0.150	**102%**
g_{Ks}	1.201	1.100	**9.2%**	0.426	0.150	**184%**
g_{CaL}	1.104	1.100	**0.4%**	0.178	0.150	**18.3%**

Table 5. Statistics summary of the estimated parameters from the Courtemanche model at 2Hz pacing frequency.

Parameter	μ	μ_{ex}	rel. err.	σ	σ_{ex}	rel. err.
g_{Na}	1.099	1.100	**0.1%**	0.151	0.150	**1.0%**
g_{K1}	1.110	1.100	**0.9%**	0.167	0.150	**11.6%**
g_{to}	1.109	1.100	**0.8%**	0.185	0.150	**23.3%**
g_{Kr}	1.064	1.100	**3.2%**	0.366	0.150	**144%**
g_{Ks}	1.263	1.100	**14.9%**	0.424	0.150	**183%**
g_{CaL}	1.132	1.100	**2.9%**	0.208	0.150	**38.4%**

Table 6. Statistics summary of the estimated parameters from the Courtemanche model (combined 1Hz+2Hz data).

Parameter	μ	μ_{ex}	rel. err.	σ	σ_{ex}	rel. err.
g_{Na}	1.098	1.100	**0.2%**	0.149	0.150	**0.4%**
g_{K1}	1.103	1.100	**0.2%**	0.166	0.150	**11.0%**
g_{to}	1.106	1.100	**0.6%**	0.165	0.150	**9.7%**
g_{Kr}	1.096	1.100	**0.4%**	0.266	0.150	**77.2%**
g_{Ks}	1.150	1.100	**4.6%**	0.424	0.150	**183%**
g_{CaL}	1.102	1.100	**0.2%**	0.161	0.150	**7.4%**

Table 7. Davies model: experimental parameter calibration and observable moment matching results.

Conductance	OMM results		Reference values		
	μ	σ	exp. calibration	literature	unit
g_{Na}	-	-	27.4	8.25	nS/pF
g_{NaL}	-	-	7.26×10^{-2}	1.1×10^{-2}	nS/pF
g_{Kr}	**0.96**	**0.03**	4.82×10^{-2}	1.39×10^{-2}	nS/pF
g_{Ks}	**1.06**	**0.12**	1.38×10^{-1}	7.47×10^{-3}	nS/pF
g_{K1}	-	-	2.91×10^{-1}	5.0×10^{-1}	nS/pF
g_{Kp}	-	-	7.93×10^{-3}	2.76×10^{-3}	nS/pF
g_{to1}	**1.03**	**0.11**	0.38	0.18	nS/pF
g_{CaL}	-	-	2.0×10^{-4}	9.7×10^{-4}	nS/pF
g_{bCa}	-	-	5.88×10^{-6}	7.98×10^{-7}	nS/pF
g_{pCa}	-	-	9.84×10^{-4}	5.75×10^{-2}	pA/pF
g_{Clb}	-	-	9.45×10^{-6}	2.25×10^{-4}	nS/pF
g_{to2}	-	-	4.54×10^{-9}	4×10^{-7}	L/F.ms
g_{NaCa}	-	-	6.95×10^{-2}	5.85	pA/pF
g_{NaK}	-	-	6.6×10^{-3}	6.19×10^{-1}	pA/pF

Table 8. Human biomarkers dataset statistics.

group		APD90	APD50	APD20	APA	RMP	dV/dt_{max}	V20
SR	representative (#163)	**324**	**142**	**5**	**93**	**-74**	**214**	**-19**
	median	319	151	5	95	-74	213	-16
	mean	317.7	138.7	7.1	94.8	-73.8	219.7	-16.1
	std	42.7	44.1	8.2	7.1	3.9	67.9	6.4
AF	representative (#131)	**213**	**105**	**38**	**98**	**-76**	**237**	**0**
	median	213	101	27	102	-77	229	-4
	mean	216.4	101.5	30.2	101.6	-76.8	232.0	-4.4
	std	34.4	27.7	17.9	7.6	3.6	70.3	10.9

Table 9. Courtemanche model: experimental parameter calibration.

Conductance	Experimentally calibrated (normalized)			Reference value	unit
	SR group	AF group	AF/SR rel. change		
g_{Na}	1.17	1.11	-5%	7.8	nS/pF
g_{K1}	0.45	1.45	**+220%**	9.0×10^{-2}	nS/pF
g_{to}	1.12	2.83×10^{-3}	**-100%**	1.65×10^{-1}	nS/pF
g_{Kr}	1.15	1.41	+23%	2.94×10^{-2}	nS/pF
g_{Ks}	1.10	1.21	+10%	1.29×10^{-1}	nS/pF
g_{CaL}	0.87	0.32	**-63%**	1.24×10^{-1}	nS/pF
g_{bNa}	1.26	1.31	+4%	6.74×10^{-4}	nS/pF
g_{bCa}	1.14	1.85	+63%	1.13×10^{-3}	nS/pF
g_{NaK}	0.78	0.80	+3%	0.6	pA/pF
g_{NaCa}	4.65×10^{-2}	2.01	×43	1.6×10^{3}	pA/pF
g_{Kur}	0.54	0.21	**-60%**	1.0	dimensionless

Table 10. Statistics summary of the estimated parameters from the Courtemanche model with experimental data (SR and AF group), normalized by the reference values.

Group	SR		AF	
Parameter	μ	σ	μ	σ
g_{Na}	1.464	0.652	1.310	0.533
g_{K1}	0.431	0.125	1.744	0.656
g_{to}	1.090	0.219	0.310	0.392
g_{Kr}	0.934	0.375	1.997	1.314

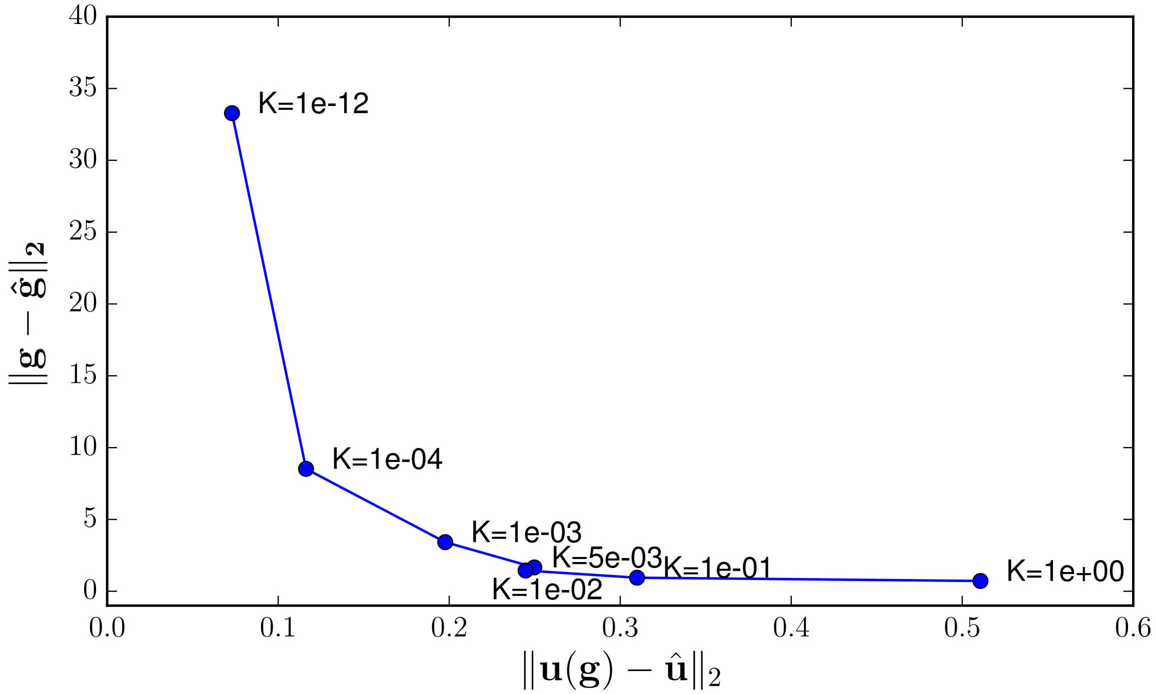

Figure 1. L-curve: calibration fitness plotted against the distance to reference conductances.

Influence of K in the calibration step We replicated the calibration procedure in Test Case 4 with the SR group representative several times by varying the regularization parameter K. In Figure 1, the fitness is plotted against the distance to the reference conductances values for different values of K. $\mathbf{g}$ denotes the conductance values estimated by the CMA-ES algorithm, $\hat{\mathbf{g}}$ are the reference values for these conductances, $\mathbf{u}(\mathbf{g})$ are the simulated biomarkers and $\hat{\mathbf{u}}$ are the experimental biomarkers to be fitted. We obtain a so-called "L-curve" which is typical of such hyper-parameter studies. We see that K=5e-3 is a reasonable choice as it is close the curve's elbow and therefore a good trade-off between goodness of fit and distance to the reference values.

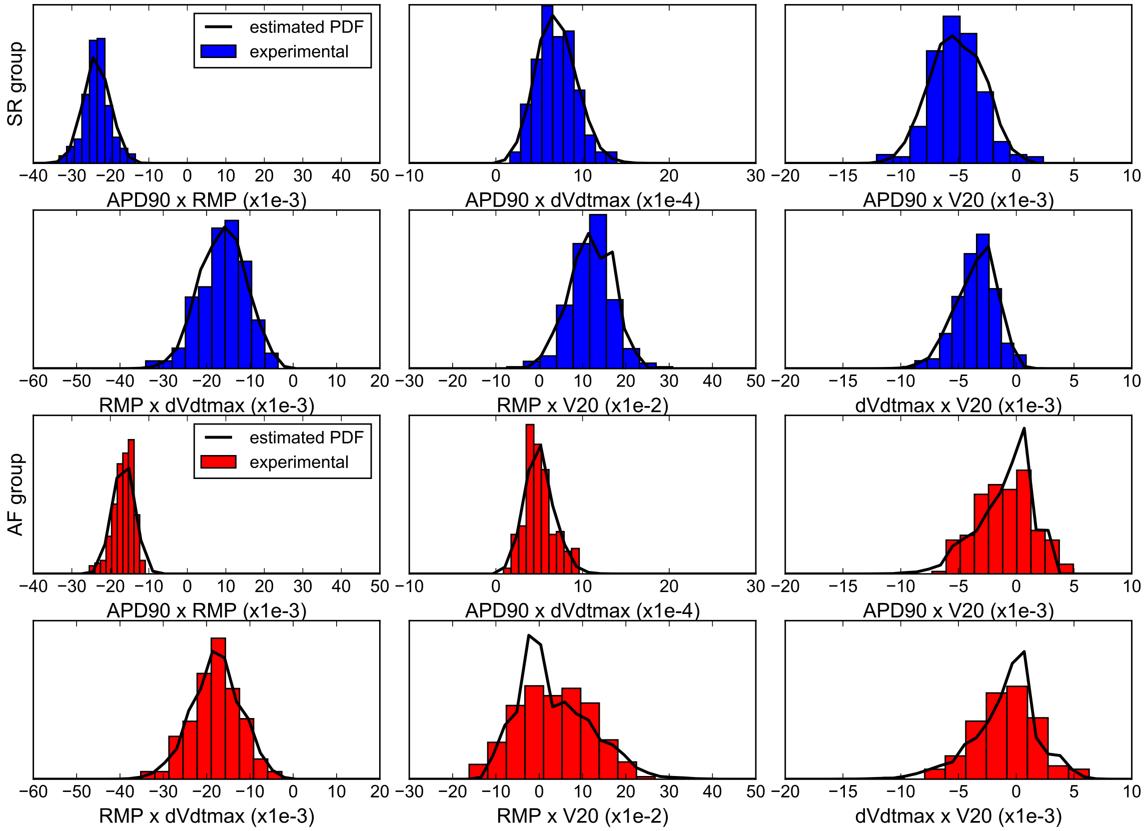

Figure 2. Normalized histograms of the experimental biomarkers pairwise products for both SR (blue) and AF (red) groups. The black solid lines correspond to the PDF of each pairwise product estimated by the observable moment matching method. Biomarkers computed from an AP. This figure is an extension of Fig.7(C) in the main article.

Appendix A: Illustration of the OMM method using the Davies model

We propose to illustrate the OMM approach using a simple test case that highlights its non-parametric nature. Indeed, the PDF is estimated point-wise in the parameter space and therefore potentially any distribution may be recovered. We propose to study a test case where one of the parameters has a uniform distribution and two of them are correlated.

For the synthetic data set, $N = 10^4$ APs were generated using the Davies model with three uncertainparameters: g_{Na}, g_{Kr}, g_{CaL}. g_{Kr} and g_{CaL} were drawn from a bivariate normal distribution of mean vector $\boldsymbol{\mu} = (1, 1)$ and of covariance matrix:

$$A = 10^{-2} \times \begin{pmatrix} 2.25 & 2.03 \\ 2.03 & 2.25 \end{pmatrix}$$

which means g_{Kr} and g_{CaL} have a standard deviation of 0.15 and a Pearson correlation coefficient of 90%. g_{Na} is sampled from an uncorrelated uniform distribution over $[0.5, 2.]$. The simulation database was built by sampling the same three parameters over the domain $\boldsymbol{\Theta} = [0.3, 2.0]^3$. $Nc = 2^{12}$ samples were drawn and the number of moments to be matched was set to $N_m = 3$. The estimated marginals are shown in Fig. 3. We also plotted the estimated correlation matrix of the three conductances in Fig. 4. The correlation between g_{Kr} and g_{CaL} is captured by the method even though it is under-estimated at 60% instead of the 90% of the true distribution.

The discrepancy observed between the true and estimated correlation would be not be observed when using Bayesian inference techniques. This is a limitation of the proposed method. In this particular case, the observable quantities could be enriched with auto-correlation quantities to be able to correctly estimate the parameters correlation.

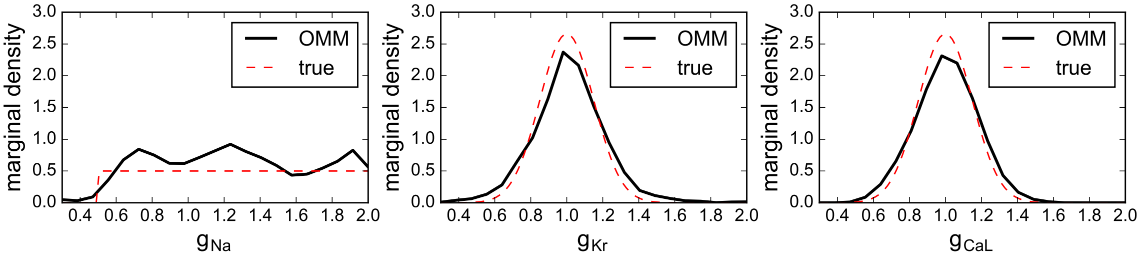

Figure 3. Conductances estimated marginal densities.

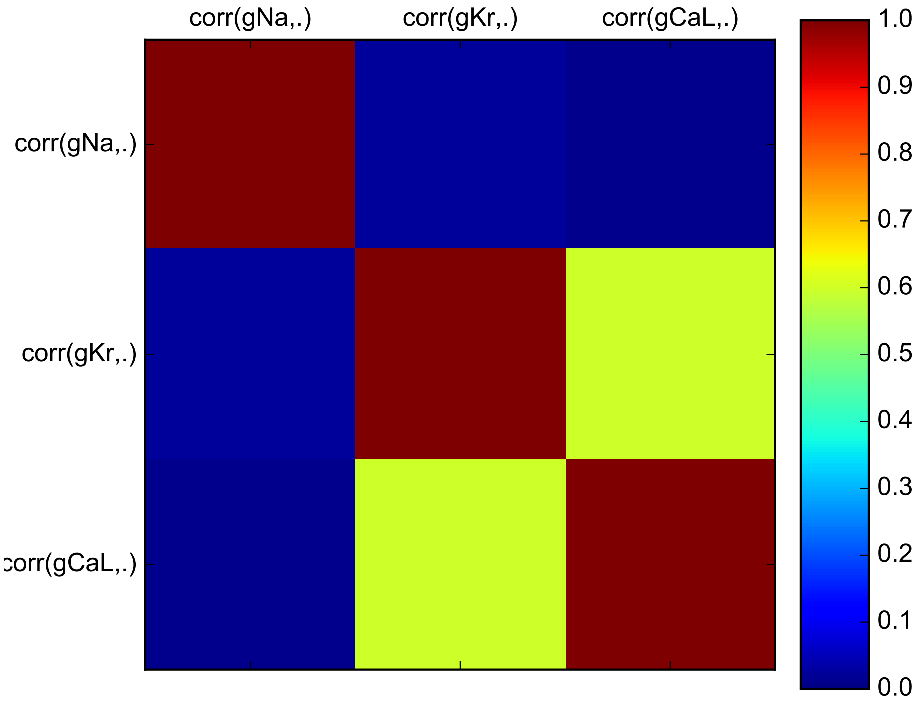

Figure 4. Estimated correlation matrix.
